# Maximum Anterior Tongue Strength and Maximum Lip Strength in Healthy Spanish Adults: A Proposal of Reference Values

**DOI:** 10.1007/s00455-024-10670-w

**Published:** 2024-01-19

**Authors:** Enrique Marín-Bernard, María Dolores Ruiz-López, Basilio Gómez-Pozo, Reyes Artacho

**Affiliations:** 1 Departament of Nutrition and Food Science, Faculty of Pharmacy, University of Granada, Granada, 18071 Spain; 2 Endocrinology and Nutrition Unit, Virgen de las Nieves University Hospital, Andalusian Health Service, Granada, 18014 Spain; 3 Biomedical Research Center, Institute of Nutrition and Food Technology “José Mataix, ” University of Granada, Granada, 18100 Spain; 4 Research Unit of the Granada-Metropolitan Health District, Andalusian Health Service, Granada, 18013 Spain

**Keywords:** Tongue, Lips, Strength, IOPI, Spanish, Adult

## Abstract

Adequate tongue and lip strengths are needed for normal speech, chewing, and swallowing development. The aim was to evaluate the influence of sex and age on maximum anterior tongue strength (MTS) and maximum lip strength (MLS) in healthy Spanish adults to establish reference values that can be used in clinical practice.

This cross-sectional study comprises 363 subjects (mean age 47.5 ± 20.7 years) distributed by sex (258 women and 105 men) and across three age groups: Young (18–39 years), middle-aged (40–59 years), and older adults (> 59 years). MTS and MLS were determined using the Iowa Oral Performance Instrument (IOPI). The mean MTS was 49.63 ± 13.81 kPa, regardless of sex, and decreased with age. The mean MLS was statistically higher for men (28.86 ± 10.88 kPa) than for women (23.37 ± 6.92 kPa, *p* = 0.001), regardless of age.

This study provides the first reference values for the standardized measurement of MTS and MLS in a healthy adult Spanish-speaking population using the IOPI.

## Introduction

Adequate tongue and lip strengths are necessary for the normal development of speech, chewing, and swallowing [1, 2]. Their determination through portable devices such as the Iowa Oral Performance Instrument (IOPI) (IOPI Medical LLC, Washington, USA) [3] is widely used today in the clinical diagnosis and treatment of dysphagia disorders [4], myofunctional therapy, sleep-disordered breathing, and in the evaluation of patients with amyotrophic lateral sclerosis [5, 6]. The reference values proposed by IOPI were established from the results obtained from eleven studies conducted in the American population and, therefore, may not be suitable for studies performed in other populations [3].

In addition to sex, age [6], and the location of the oral cavity where these strengths are measured [7], several studies indicate that ethnicity and language influence the values of tongue and lip strengths in healthy populations and these values must be used as a reference for comparation with the values obtained in the diagnosis or therapies used in various conditions [2, 8].

However, data on the influence of ethnicity and language on tongue and lip strengths in healthy populations are scarce. Most of the data come from studies conducted in Asia and the United States, and only two studies have been conducted in Europe, including only data on older populations [9]. To date, there are no specific data available on tongue and lip strength in the Spanish population. It is of great interest to establish these reference values in a Spanish-speaking community, given that Spanish is the mother tongue of 496 million people and the second most widely spoken language in the world after Mandarin Chinese [10]. Spanish is a Romance language with phonetic similarities to other European languages such as French, Portuguese, Italian, and Romanian, among others, while English is a Germanic language related to German or Danish [11].

Therefore, the objective of the present study was to evaluate the effects of sex and age on the maximum anterior tongue strength (MTS) and the maximum lip strength (MLS), measured by IOPI, in healthy Spanish adults to establish reference values that can be used in clinical practice in this population.

## Materials and Methods

### Study Design

A cross-sectional study was conducted on individuals of both sexes over 18 years of age and whose native language is Spanish. A non-probability sampling of patients and staff of the Virgen de las Nieves University Hospital in Granada and community dwelling adults from various healthcare and social health centers in Granada was performed.

Previous research studies on the reliability of the measurement using the IOPI Model 2.3 [3] were considered to estimate the sample size. The sample size calculation was based on the method suggested by Wolak et al. [12] For this purpose, the library for the intraclass correlation coefficient (ICC) developed by the authors was used for the computer program R version 2.3.0 [13]. A sensitivity analysis was performed, assuming ICC values between 0.4 and 0.9 and between 2 and 6 repetitions of the measurement. Therefore, a sample size of 200 participants would be sufficient for an accurate estimate [12].

### Inclusion and Exclusion Criteria

Individuals with no health problems that would prevent them from performing the required measurements and who voluntarily signed the informed consent were included in the study. All subjects with a clinical diagnosis of oropharyngeal dysphagia or speech disorders, neurological and genetic disorders with alteration of consciousness, breathing, chewing, cognitive-behavioral disorder, tumors of the respiratory tract, upper digestive tract, and head, recent surgery in the respiratory tract, upper digestive tract, and head, anatomical or physiological alteration of the tongue or salivary glands, opioid analgesia treatments, sedatives (with active ingredients of half-life longer than six hours), upper respiratory and digestive tract infections, and respiratory pattern alterations (asthma, rhinitis or allergies) were excluded from the study. These exclusion criteria were confirmed by the clinical history of the patients and by a personal interview with the community dwelling adults. Data were gathered by a qualified dietician-nutricionist (EM).

### Ethical Considerations

The protocol for this study was approved by the Research Ethics Committee of the University of Granada (nº 149/CEIH/2016). All study participants signed the informed consent. This study was performed in accordance with the Declaration of Helsinki [14].

### Instrumentation and Procedures

The IOPI Model 2.3 was used to measure tongue strength and lip strength. This portable pressure instrument consists of an air-filled bulb connected to the device through an 11 cm tube. The bulb is 3.5 cm long and 4.5 cm in diameter. The device displays pressures in kilopascals (kPa) on its LCD screen. Material preparation and the protocol used for strength measurements were according to the manufacturer’s instructions [3].

Each participant was shown an image of the correct bulb placement and was given instructions before the measurements. All measurements were performed with the participants sitting upright and instructing them to press the bulb with maximum effort for two seconds and receiving verbal encouragement from the researcher saying: “squeeze, squeeze, squeeze!”. Each measurement was performed in triplicate with a 60-seconds rest between repetitions. For each participant, the highest value of the strength exerted was recorded from the three measurements performed [15] for both the MTS and the MLS.

**Fig. 1:**
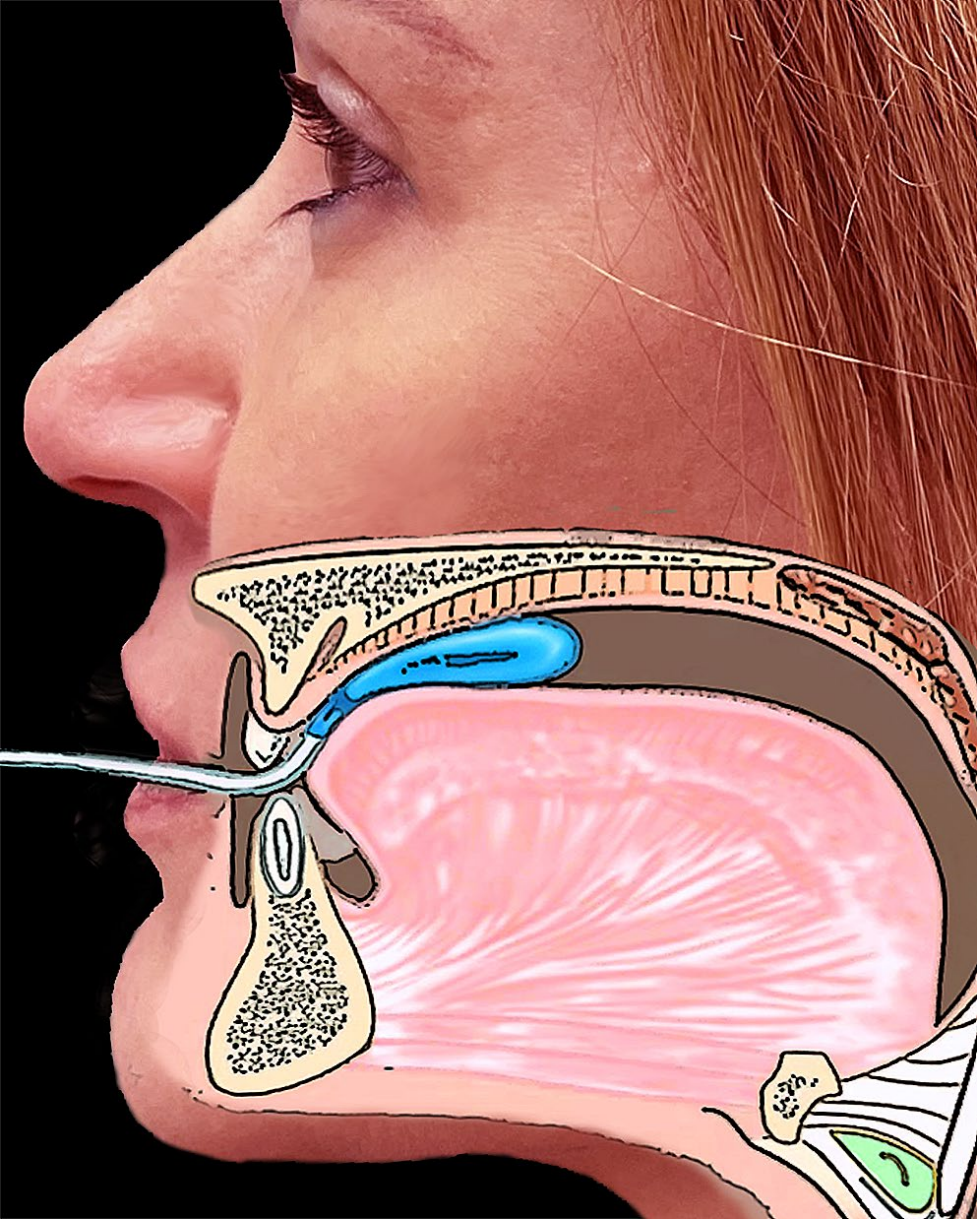
Schematic representation of the location of the bulb in the center of the tongue, just behind the incisors

The bulb was placed in the center of the tongue, just behind the incisors, to measure the MTS. Participants were instructed to press the bulb against the anterior palate (roof of the mouth) with the tongue as hard as possible, as described above [3] (Fig. 1).

**Fig. 2:**
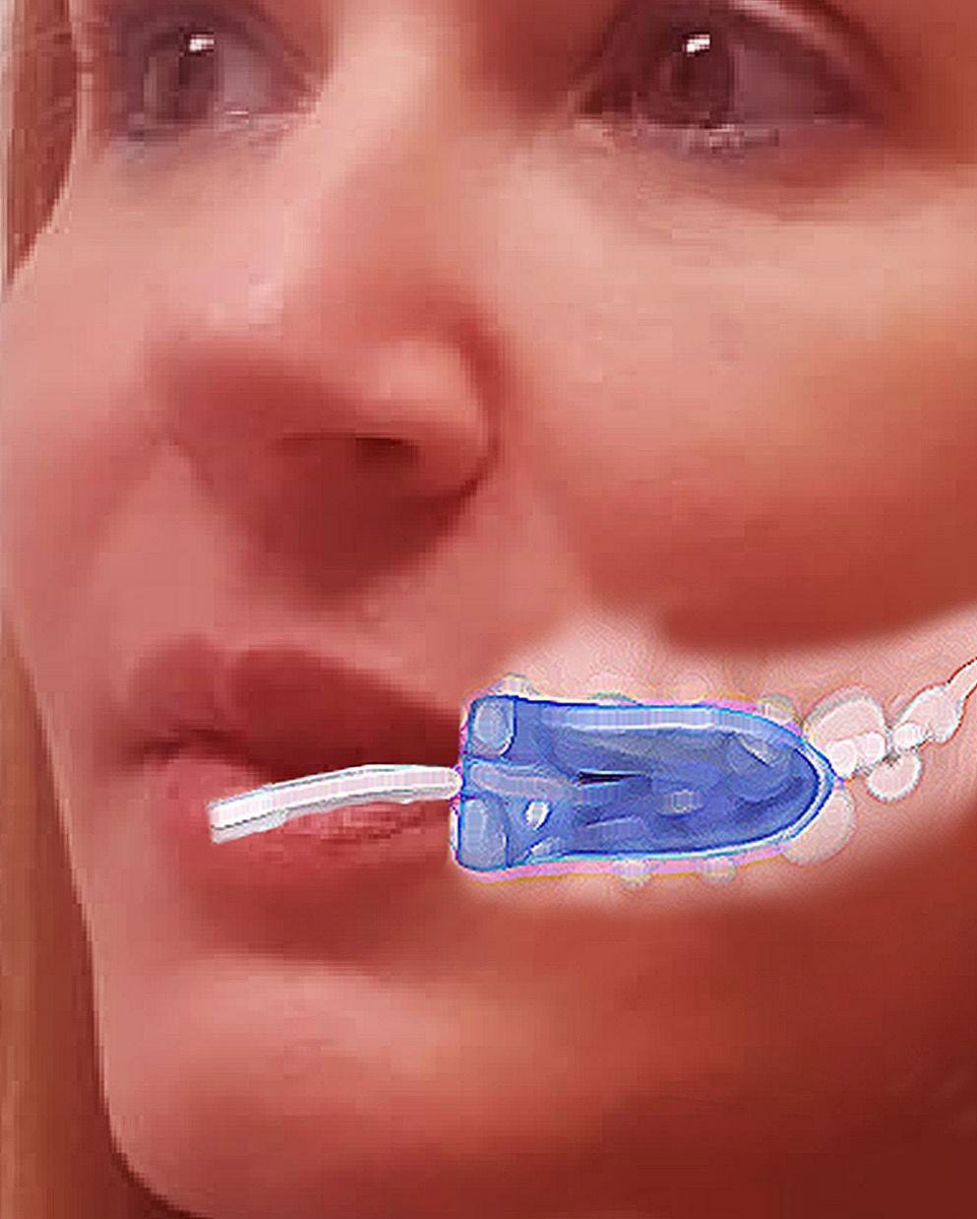
Schematic representation of the location of the bulb inside the left cheek, just lateral to the corner of the mouth

MLS was measured as a function of the pressure generated by the orbicular muscle of the mouth (musculus orbicularis oris). The bulb was located inside the left cheek, just lateral to the corner of the mouth. Participants were instructed to squeeze the bulb against the oral surface of the teeth by squeezing their lips as hard as possible, as described above [3] (Fig. 2).

### Statistical Analysis

The normal distribution for the variables studied (MTS, MLS, sex and age) was analyzed using the Shapiro-Wilk test. Descriptive statistics (means and 95% confidence intervals (CI) of the mean and minimum and maximum values) were calculated for MTS and MLS, the total sample, and according to sex and age groups. The linear Pearson´s correlation coefficient (r) for MTS and age was determined in men, and Spearman´s correlation coefficient for MTS in both sex and in women for MLS. The Mann-Whitney test was used to analyze the sex difference in MTS and MLS, as well as the difference between the three established age groups. For the MTS and MLS, pairwise comparisons were performed for the age groups established independently in women and men. The Krusal-Wallis test for independent samples was used in the women’s group. The Bonferroni test for multiple comparisons was used for MTS and men using the ANOVA technique, and the Krusal-Wallis test for independent samples for pairwise comparisons was used for MLS. Data were analyzed using SPSS software version 26 (IBM, Armonk, NY, USA). UU.).

## Results

The sample consisted of 363 participants (258 women and 105 men) with a mean age of 47.5 ± 20.7 years and was divided into three age groups: Young (18–39 years), middle-aged (40–59 years) and older adults (> 59 years) (Table 1).

**Table 1:** Participant characteristics

Groups by age	Total			Women			Men		
	n	%	Middle Ages ± DS (years)	n	%	Middle Ages ± DS (years)	n	%	Middle Ages ± DS (years)
Young adults	136	37.5	25.2 ± 5.7	100	73.5	24.6 ± 5.7	36	26.5	26.8 ± 5.6
Middle-aged adults	123	33.9	50.4 ± 5.4	100	81.3	50.7 ± 5.3	23	18.7	48.8 ± 5.9
Older adults	104	28.7	73.4 ± 8.9	58	55.5	71.9 ± 9.01	46	44.2	75.4 ± 8.6
Total	363		47.5 ± 20.7	258	71.07	45.3 ± 19.4	105	28.9	52.9 ± 22.6

n = number of participants; Young adults (18–39 years); Middle-aged adults (40–59 years); Older adults = (> 59 years)

### MTS: Influence of Sex and Age

**Fig. 3:**
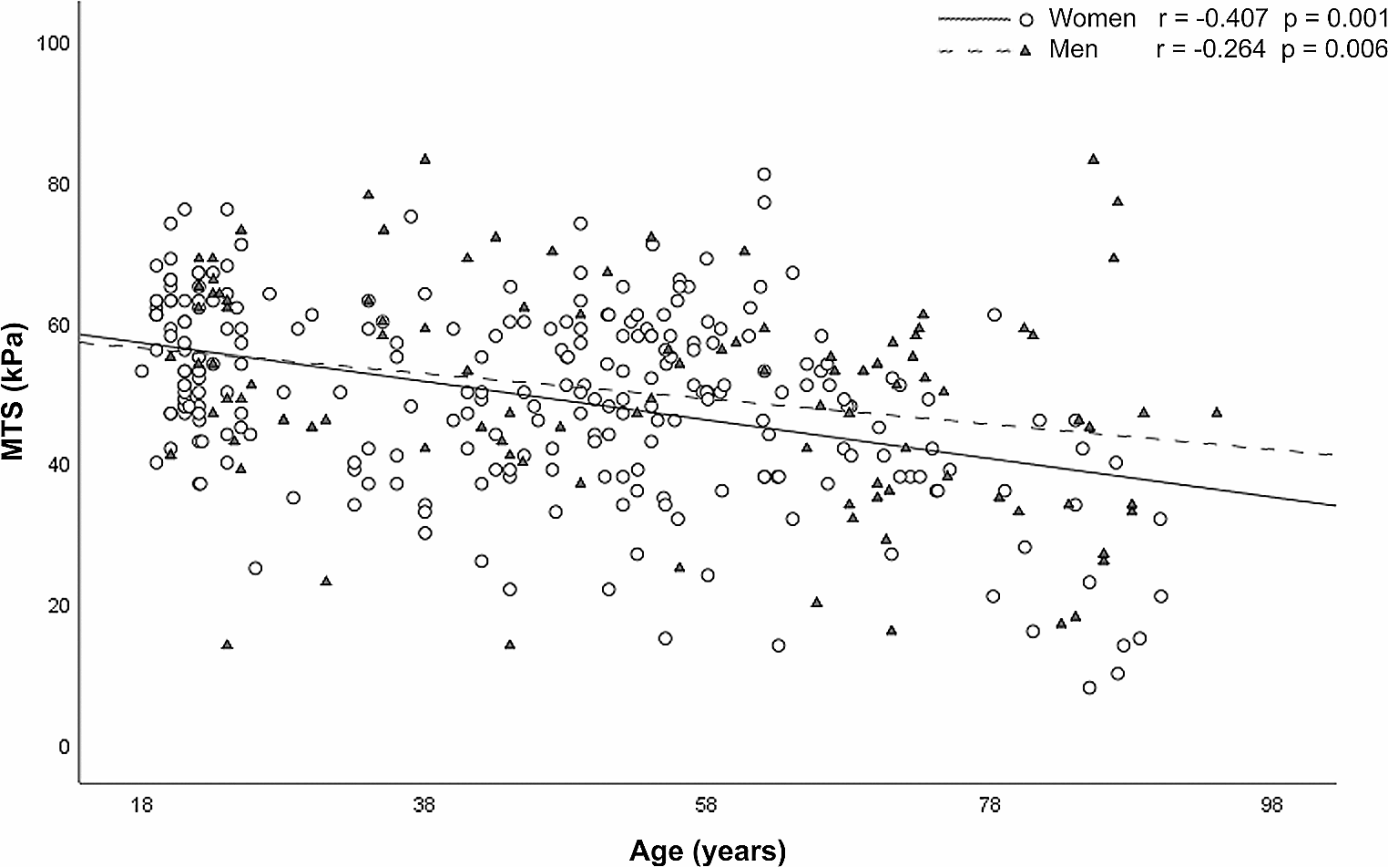
Linear regression analysis between maximum tongue strength (MTS) (kPa) and age in men (π) and women (ϒ)

**Fig. 4:**
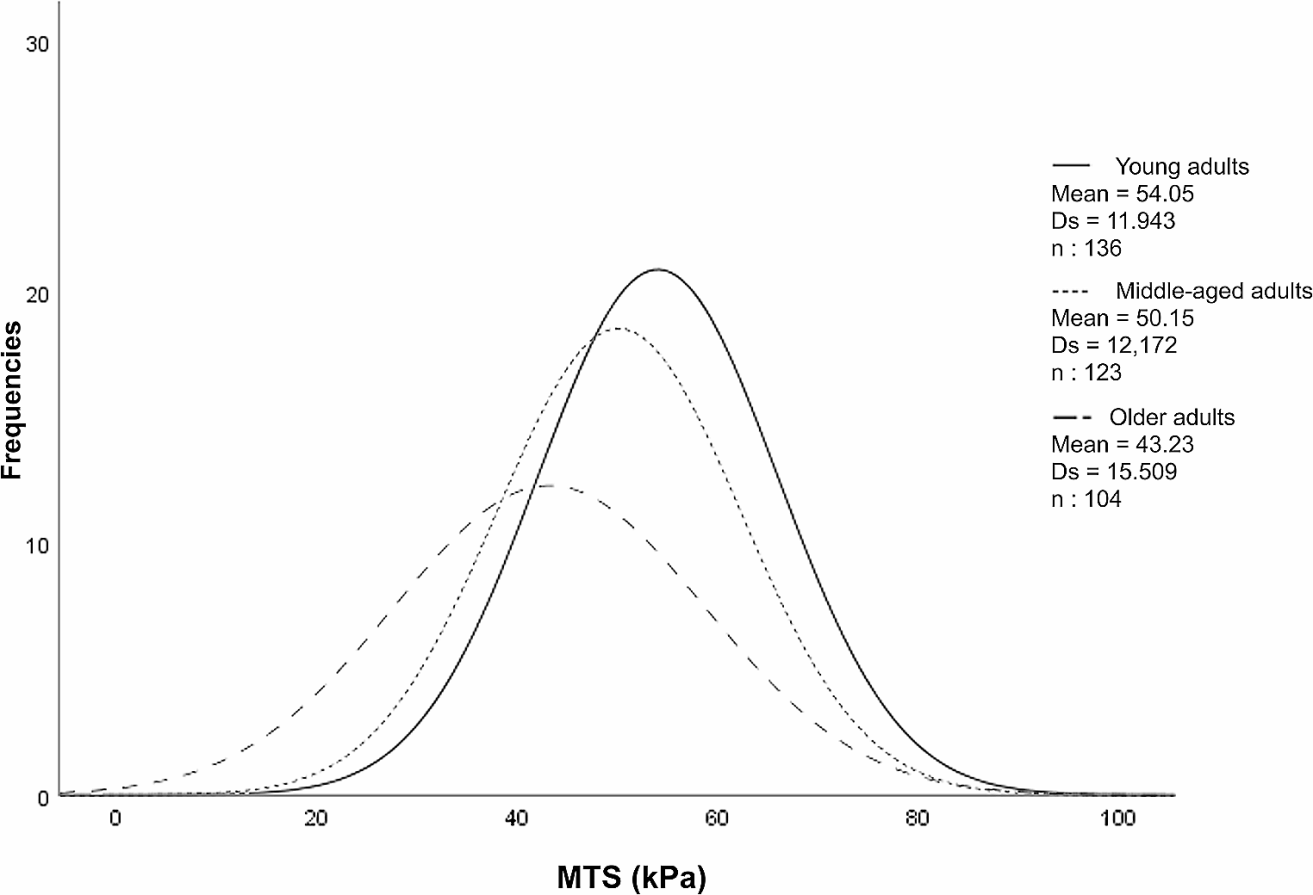
Density curves of the maximum tongue strength (MTS) (kPa) based on the established age groups

No statistically significant differences were found in MTS values by sex in the total sample (49.63 ± 13.81 kPa, *p* = 0.797) or when considering intra-age groups, regardless of sex: Young (54.05 ± 11.94 kPa, *p* = 0.617), middle-aged (50.15 ± 12.17 kPa, *p* = 0.604), and older adults (43.23 ± 15.5 kPa, *p* = 0.226) (Table 2). MTS values decrease with age. Linear regression analysis revealed a statistically significant correlation between MTS and age in both men (*r* = -0.264; *p* = 0.006) and women (*r* = -0.407; *p* = 0.001) (Fig. 3). Statistically significant differences were found between the three age groups in women (*p* = 0.001) and in men (*p* = 0.015) (Table 2). Post hoc tests indicated significant differences between young and older adults (*p* = 0.001) and between young and middle-aged adults (*p* = 0.001) in both the total sample and when considering women. In the case of men, significant differences were found only between young and older adults (*p* = 0.013). Figure 4 shows the density curves of the MTS based on the established age groups.

**Table 2:** MTS (kPa) y MLS (kPa) grouped by sex and age

Groups by age	All						Women						Men					
	X ± DS	95% CI		Min	Max	*p* value	X ± DS	95% CI		Min	Max	*p* value	X ± DS	95% CI		Min	Max	*p* value
		Lower	Upper					Lower	Upper					Lower	Upper			
*MTS (kPa)*																		
Young adults	54.05 ± 11.94	52.03	56.08	14	83	0.617	53.7 ± 10.98	51.52	55.88	25	76	0.001	55.03 ± 14.41	50.15	59.91	14	83	0.015
Middle-aged adults	50.15 ± 12.17	47.98	52.33	14	74	0.604	49.87 ± 11.57	47.57	52.17	15	74		51.39 ± 14.73	45.02	57.76	14	72	
Older adults	43.23 ± 15.5	40.21	46.25	8	83	0.226	41.59 ± 15.48	37.51	45.66	8	81		45.30 ± 15.45	40.71	49.89	16	83	
Total	49.63 ± 13.81	48.21	51.06	5	83	0.797	49.49 ± 13.12	47.37	51.61	8	81		49.97 ± 1.43	46.02	53.92	14	83	
*MLS (kPa)*																		
Young adults	24.81 ± 5.54	23.87	25.75	13	40	0.001	23.56 ± 4.8	22.61	24.51	13	38	0.318	28.28 ± 6.02	26.24	30.32	16	40	0.603
Middle-aged adults	24.63 ± 6.93	23.40	25.87	7	57	0.001	23.54 ± 6.46	22.26	24.82	7	57		29.39 ± 7.02	26.35	32.43	15	41	
Older adults	25.54 ± 12.78	23.05	28.03	2	85	0.012	22.76 ± 10.15	20.09	25.43	2	73		29.04 ± 14.87	24.63	33.46	4	85	
Total	24.96 ± 8.61	24.07	25.85	2	85	0.001	23.37 ± 6.92	22.52	24.22	2	73		28.86 ± 10.88	26.75	30.96	4	85	

MTS: maximum anterior tongue strength; MLS: maximum lip strength; Young adults (18–39 years); Middle-aged adults (40–59 years); Older adults (> 59 years)

### MLS: Influence of Sex and Age

**Fig. 5:**
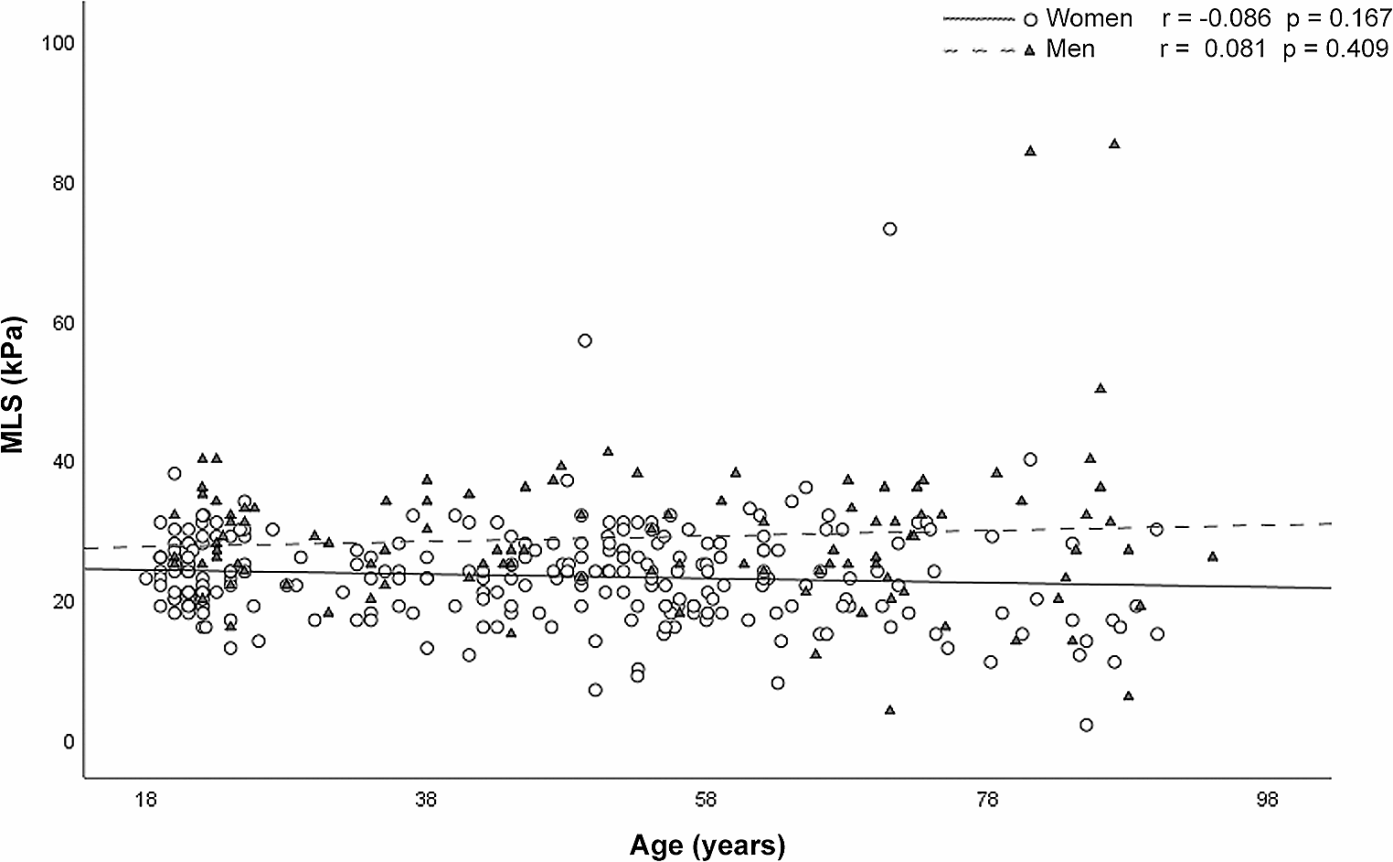
Linear regression analysis between maximum lip strength (MLS) (kPa) and age in men (π) and women (ϒ)

**Fig. 6:**
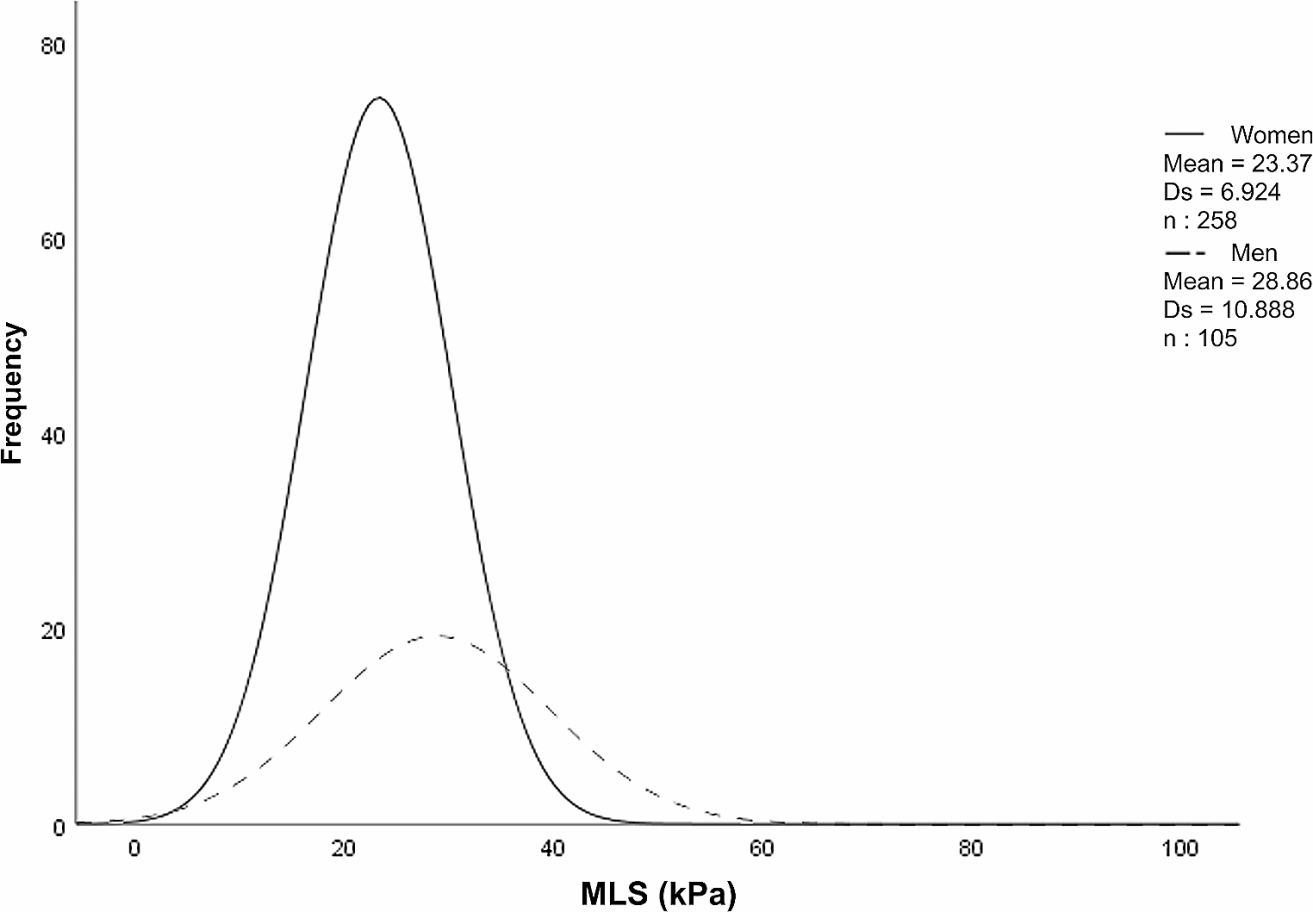
Density curves of maximum lip strength (MLS) (kPa) in men and women The six images of the article that have been created and processed with the software Adobe Photochop CS3 Extended versión 10.0

The mean MLS was 24.96 ± 8.61 kPa; the MLS was statistically higher in men (28.86 ± 10.88 kPa) than in women (23.37 ± 6.92 kPa, *p* = 0.001) in the total sample and for all age groups. Linear regression analysis revealed no statistically significant correlation between MLS and age in men (*r* = 0.081; *p* = 0.409) or women (*r* = -0.086; *p* = 0.167) (Fig. 5). MLS values are independent of age groups, both in the total sample and between age groups in women (*p* = 0.318) and in men (*p* = 0.603). Post hoc tests indicate no significant differences between the three groups compared by age and sex (*p* = 1). Figure 6 shows the density curves of MLS in both sexes.

Table 3 shows the proposed reference values for the Spanish population (expressed in percentiles) of MTS according to age groups and MLS according to sex.

**Table 3:** Normal values for MTS and MLS

Percentiles	1%	5%	10%	20%	25%	50%
*Groups by age*	*MTS (kPa)*					
Young adults	17.33	34	39	44	46.25	54.5
Middle-aged adults	14.24	25.2	34.4	39.8	43	50
Older adults	8.10	15.25	20.5	32	34	43
*Sex*	*MLS (kPa)*					
Women	4.12	14.30	18	22	23.5	27
Men	7.59	13.95	16	18	19	23

MTS: maximum anterior tongue strength; MLS: maximum lip strength; Young adults (18–39 years); Middle-aged adults (40–59 years); Older adults = (> 59 years)

## Discussion

In the present study, we evaluated the influence of sex and age on MTS and MLS in healthy adults whose native language is Spanish to provide reference values for the Spanish population because, as far as we know, no data are available for the scientific community. Only one of the studies aiming to provide reference data of the MTS and the MLS, using the IOPI device and considering the ethnicity or the language [7, 8, 16–21] has been conducted in Europe, specifically in Belgium [7].

Our results indicate that MTS decreases with age regardless of sex, and it is within the range considered adequate for a healthy adult population (43–78 kPa) [6].

The mean MTS in this study can be considered as similar to that found in the Belgian population [7] (5 kPa difference) but in that study participants spoke Dutch, a Germanic language while Spanish is a Romance language, as previously mentioned [11] and the data provided do not allow any conclusions to be drawn in this respect. However, our results are lower than those found in the North American population [16–18] (8–33 kPa difference) and in the Chinese population [19] (7 kPa difference). This issue reinforces the need to carry out studies which consider regional standard values and cross-national surveys of tongue strength taking into account various perspectives, such as age, gender, race and nationality [19] and therefore language.

The influence of sex is complex [9]. In consistency with other studies [7, 16, 18, 19], we found no significant sex differences. In studies reporting differences conducted in Taiwan [8], and United States [17] men showed statistically higher values than women. Another study conducted in Koreans makes no mention of sex [20]. The results of the systematic review and meta-analysis of Arakawa et al. [9] suggest no influence of sex when considering individuals older than 60 (*p* = 0.282); in contrast, sex is a variable to consider in individuals younger than that age (*p* = 0.004).

Our results indicate that MTS decreases with age, in both men and women, which is consistent with previous studies such as in the Belgian population [7], in the Chinese population [8] and in the USA population [16]. When classifying the sample by age groups [3], our results indicate a lower MTS in the older adults than in the young adults in the total sample, women, and men. In addition, significant differences were found between middle-aged adults and young adults in the total sample and women. Other studies [16, 18] also show a lower MTS in the elderly group compared with the middle-aged adults, although MTS values in the middle-aged adults are higher than those in the young adults. However, the influence of age on MTS in the Korean population is not clear [20, 21].

Healthy aging is related with changes in the musculature of oro-facial structures such as a reduction in the amount of muscle fibers and motor units and an increase of intramuscular fat. Consequently, there is a decrease in the muscle mass and the strength of the tongue and lips, associated with sarcopenia [22]. Today, the number of studies presenting evidence and association between a decrease in tongue strength and dysphagia and sarcopenia in the elderly population is increasing [23–25]. Sarcopenic dysphagia is defined as a swallowing disorder due to sarcopenia involving the whole-body skeletal muscles and swallowing muscles [26]. Its diagnostic algorithm, developed by the Working Group on Sarcopenic Dysphagia, considers tongue strength to measure the strength of swallowing muscles [27]. It is therefore important to establish appropriate cut-off points of tongue strength for the older population that can be used in the clinical practice.

The lips, constitute an articulatory organ and an important communication tool in expressing emotion an in mastication and pronunciation Studies providing data on lip strength in a healthy adult population are very scarce and, unlike MTS, do not allow to establish an adequate range of MLS. There is also insufficient data showing a decrease in MLS with healthy aging and most studies do establish a greater MLS in men than in women [28]. Our results indicate that MLS is higher in men than in women (4 kPa difference) and independent of age. This is consistent with the study performed in the American population [18], with values of 33.8 ± 15.1 kPa in men and 22.4 ± 7.5 kPa in women (*p* < 0.001). This value obtained for men is higher than that found in our study (11 kPa difference). Other study providing data on MLS was performed in the Korean population [21], whose values were lowers than in our study (11 kPa difference), although no differences based on sex or age groups were found. In that country the study conducted by Park et al. [2], established a significant age-related decrease of MLS. It is important to point out that although these studies [18, 21] used the IOPI device, the lip strength was determined by interspersing the bulb between two wood depressors, and therefore, the values obtained cannot be compared with those obtained in our study. It is therefore difficult to make an interpretation of the results obtained in different studies.

The availability of national reference values for MTS and MLS in healthy populations is a prerequisite for using IOPI in clinical practice [7]. The lack of standardization protocols such as sample size, mean age, established age groups, or not referencing the language can distort the results of the different studies. The sample size of the studies analyzed ranges from less than 100 [16, 18] individuals to 420 individuals [7]. In this study, a sample size of 363 individuals has been established depending on the measurement reliability of the IOPI Model 2.3 [3]. The age range is between 18 years in the present study and the United States study [18], up to 96 years [7], and the mean age is between 42.34 ± 20.3 years [18] and 47.5 ± 20.7 years in this study.

In the present study, in consistency with other studies [16, 18, 21], the sample has been divided according to the age groups proposed by the IOPI (young, middle-aged, and older adults) [3]. Other studies establish 10-year age groups [7, 8], including a group for over 80 [7] or an age group for over 71 [8] or only 2 age groups (young adults and elders) [20]. Regarding language, only the studies conducted in the Belgian population [7] and Taiwanese population [8] specify the language; Dutch and Taiwanese native of Taiwanese descent speaking Taiwanese and Mandarin, respectively.

Among the strengths of the present study, it is worth mentioning that it is the first study providing data on the standardized measurement of MTS and MLS in a healthy Spanish population. The sample size is large, and exclusion criteria have been carefully selected. In addition, the IOPI is considered a useful tool to successfully measure tongue and lip strength, not only in healthy subjects but also in patients with different diseases, and also allows detection of the productive effects of language training in both healthy and diseased subjects [4]. As limitations, it should be noted that other factors that might have influenced the values obtained have not been considered, such as nutritional status or physical activity, and that the sample selection has been conducted exclusively in the province of Granada.

## Conclusions

The present study provides the first reference values for the standardized measurement of MTS and MLS in a healthy Spanish population using the IOPI. These values may be useful in clinical practice. MTS is age-dependent and independent of sex, while MLS is higher in men than in women but independent of age.

## Data Availability

All data generated or analysed during this study are included in this published article. Besides, data will be available on razonable request.
